# Human-to-Human Transmission of Monkeypox Virus, United Kingdom, October 2018

**DOI:** 10.3201/eid2604.191164

**Published:** 2020-04

**Authors:** Aisling Vaughan, Emma Aarons, John Astbury, Tim Brooks, Meera Chand, Peter Flegg, Angela Hardman, Nick Harper, Richard Jarvis, Sharon Mawdsley, Mark McGivern, Dilys Morgan, Gwyn Morris, Grainne Nixon, Catherine O’Connor, Ruth Palmer, Nick Phin, D. Ashley Price, Katherine Russell, Bengu Said, Matthias L. Schmid, Roberto Vivancos, Amanda Walsh, William Welfare, Jennifer Wilburn, Jake Dunning

**Affiliations:** Public Health England, London, UK (A. Vaughan, E. Aarons, J. Astbury, T. Brooks, M. Chand, A. Hardman, R. Jarvis, M. McGivern, D. Morgan. G. Morris, G. Nixon, C. O’Connor, N. Phin, K. Russell, B. Said, R. Vivancos, A. Walsh, W. Welfare, J. Wilburn, J. Dunning);; NIHR Health Protection Research Unit in Emerging and Zoonotic Infections, London (A. Vaughan, T. Brooks, D. Morgan, R. Vivancos, J. Dunning);; Blackpool Teaching Hospitals NHS Foundation Trust, Blackpool, UK (P. Flegg, N. Harper, S. Mawdsley, R. Palmer);; The Newcastle Upon Tyne Hospitals NHS Foundation Trust, Newcastle Upon Tyne, UK (D.A. Price, M.L. Schmid)

**Keywords:** Monkeypox, monkeypox virus, orthopoxvirus, transmission, nosocomial, human, viruses, zoonoses, United Kingdom

## Abstract

In September 2018, monkeypox virus was transmitted from a patient to a healthcare worker in the United Kingdom. Transmission was probably through contact with contaminated bedding. Infection control precautions for contacts (vaccination, daily monitoring, staying home from work) were implemented. Of 134 potential contacts, 4 became ill; all patients survived.

Monkeypox is a reemerging zoonosis caused by *Monkeypox virus* (MPXV), a member of the genus *Orthopoxvirus*. MPXV is related to *Variola virus*, the causative agent of smallpox. Although infections with these 2 viruses share many clinical features, monkeypox is generally less severe than smallpox (*1*). Among unvaccinated persons, the monkeypox case-fatality rate can be up to 10%, although case-fatality rates are lower for infection with the West African than the Central African clade of MPXV (*2*). In recent years, the number of cases and geographic spread of monkeypox have been increasing, possibly because of waning immunity to smallpox (*3*–*5*). Before 2018, the only human cases of monkeypox outside Africa occurred in the United States in 2003; that outbreak was associated with rodents imported from Ghana, and human-to-human transmission did not occur (*6*).

In September 2018, Public Health England (PHE) was notified of 2 unrelated cases of monkeypox affecting travelers who had recently returned from Nigeria (*7*). We describe transmission of monkeypox virus from the second of these cases to a healthcare worker (HCW) and the public health measures implemented to prevent further cases.

## The Cases

On September 6, 2018, a man with a maculopapular rash, fever, lymphadenopathy, and a 1-week history of feeling generally unwell (patient 2) sought care at a hospital in England (*7*). He was admitted to a single-occupancy room in the acute medical unit. The staff attending the patient wore standard personal protective equipment (PPE), consisting of disposable aprons and gloves. Because a travel-associated infection was considered possible, patient 2 was transferred to an isolation room on September 7, 2018.

Three days later, a clinical diagnosis of suspected monkeypox was made, and infection prevention and control precautions for a high-consequence infectious disease (HCID) were implemented (e.g., enhanced PPE consisting of disposable gown, disposable gloves, filtering facepiece 3 respirator, and face shield or goggles). The patient was transferred to an Airborne HCID Treatment Centre, and monkeypox was confirmed by laboratory testing at PHE (*7*).

Although the risk to the public was considered to be very low, a precautionary approach was adopted. Possible hospital and community contacts of patient 2 were identified and assessed for risk (Table). Because smallpox vaccines provide some cross-protection against monkeypox (*8*,*9*), a single dose of the third-generation smallpox vaccine, Imvanex (modified vaccinia Ankara; Bavarian Nordic, http://www.bavarian-nordic.com), was offered as postexposure prophylaxis (an off-label indication) to contacts at intermediate and high risk. The target vaccination window for these contacts was within 4 days of exposure, up to a maximum of 14 days from exposure. In addition, preexposure prophylaxis with Imvanex (single dose) was offered to HCID staff involved in the care of confirmed case-patients.

**Table:** Public Health England risk assessment and public health recommendations for persons potentially exposed to 2 patients with monkeypox, United Kingdom, 2018*

Risk group	Description	Public health surveillance	Postexposure vaccination with Imvanex	No. persons in risk group†	No. (%) persons in risk group who received postexposure vaccination†
No risk	No known contact (direct or indirect) with a symptomatic monkeypox case-patient‡ OR Laboratory staff handling specimens from a monkeypox case-patient, in a laboratory conforming to UK laboratory standards§	None	Not recommended	Not applicable	0
Low	HCW involved in care of monkeypox case-patient while wearing appropriate PPE (with no known breaches) for all contact episodes OR HCW involved in care of monkeypox case-patient while not wearing appropriate PPE for all contact episodes but not within 1 m of case-patient and with no direct contact with body fluids or potentially infectious material OR Community contact not within 1 m of case-patient	Passive¶	Not recommended	158	0
Intermediate	Intact skin-only contact with a symptomatic (with rash) monkeypox case-patient, their body fluids, or potentially infectious material# or contaminated fomite OR No direct contact but within 1 m of symptomatic monkeypox case-patient without wearing appropriate PPE (including disposable FFP3 respirator or equivalent)	Active#	Vaccination may be considered	125	84 (67)
High	Direct exposure of broken skin or mucous membranes to monkeypox symptomatic case-patient, patient’s body fluids, or potentially infectious material** (including clothing or bedding) without wearing appropriate PPE (including disposable FFP3 respiratory or equivalent). Exposure includes inhalation of respiratory droplets or material from scabs from cleaning rooms where a monkeypox case-patient has stayed, mucosal exposure to splashes, penetrating injury from used sharps, device or through contaminated gloves or clothing	Active#	Vaccination recommended	5	5 (100)

*Imvanex (modified vaccinia Ankara, Bavarian Nordic, http://www.bavarian-nordic.com) was approved by the European Medicines Agency in July 2013 for active immunization against smallpox in adults. Jynneos (modified vaccinia Ankara; Bavarian Nordic) was approved by the US Food and Drug Administration in September 2019 for the prevention of smallpox and monkeypox disease in adults >18 y of age determined to be at high risk for smallpox or monkeypox infection. FFP3, filtering facepiece 3; HCW, healthcare worker; PHE, Public Health England; PPE, personal protective equipment. †For patients 2 and 3 combined. ‡Case-patients are considered potentially infectious 24 h before the onset of rash. §See http://www.hse.gov.uk/pubns/books/clinical-laboratories.htm. ¶A person requiring passive surveillance is given information about monkeypox and what to do if illness develops. #A person requiring active surveillance is given information about monkeypox and instructed to report health status daily to PHE, regardless of symptoms, for 21 d from the date of most recent exposure, and to report any illness immediately. In addition, HCWs with high-risk exposures are to be excluded from work for 21 d after the most recent exposure (note this recommendation was introduced after diagnosis of the third case-patient). **Potentially infectious biological material consists of skin lesions and detached scabs.

For 3 HCWs who had been assessed for risk, including patient 3 (a healthcare assistant), the same single-exposure risk was identified: >1 episode of close contact with the bedding and clothing of patient 2 before monkeypox was diagnosed. No breaches of standard PPE were identified. All 3 staff members were classified as high-risk contacts and were placed under active surveillance and offered postexposure vaccination. Patient 3 was vaccinated against smallpox on September 14, which was 5 days after the most recent exposure and possibly 6 or 7 days after the earliest exposure to patient 2. Patient 3 had not previously received smallpox vaccine.

On September 22, while off duty, patient 3 noticed a small number of facial lesions and stayed home for the next 2 days but did not report illness to PHE. On September 24, patient 3 sought care with a general practitioner for headache, sore throat, skin lesions on the chin, earache, and eye pain. Patient 3 then reported the illness to PHE. 

The general practitioner discussed the case with PHE and provided images of the skin lesions, which were consistent with monkeypox. Further medical assessment of patient 3 at the local hospital was arranged. After assessment and collection of diagnostic specimens, patient 3 remained isolated at home. On September 25, monkeypox was confirmed by PCR testing of multiple sample types, and patient 3 was admitted to an Airborne HCID Treatment Centre.

A total of 134 possible contacts of patient 3 were identified, including staff and patients on the ward where patient 3 worked, family and community contacts, and staff and patients at the general practitioner’s office where patient 3 had sought care. Patient 3 had not been working when rash was present; however, as a precaution, all those who had had contact with patient 3 during the 24 hours before onset of the rash (i.e., on September 21) were monitored. Postexposure vaccination was offered to eligible contacts at intermediate and high risk (Table). As an extra precautionary measure, active monitoring, with daily reporting of presence or absence of signs or symptoms, was extended to all outstanding contacts of patient 2 and all new contacts of patient 3. In addition, HCW contacts at high risk were instructed not to attend work for 21 days from the most recent exposure (the incubation period for monkeypox is 5–21 days) (*10*).

A total of 4 contacts of patient 3 became ill within the incubation period and required medical assessment. No further cases of monkeypox were identified in relation to this incident and, after clinical improvement, patient 3 was discharged on October 29, 2018.

## Conclusions

Cases of human monkeypox outside Africa are rare; in the United Kingdom, the likelihood of travel-associated monkeypox cases is low (*10*–*12*). To our knowledge, human-to-human transmission of monkeypox outside Africa has not been reported, and human-to-human transmission of the West African clade has been reported for Nigeria only (*4*). Such transmission may occur through close contact with skin lesions of an infected person, via fomites, or by exposure to large respiratory droplets during face-to-face contact (*1*). The transmission reported here occurred from a patient with a travel-associated case to an HCW. The only exposure risk identified during assessment of patient 3 was the changing of potentially contaminated bedding, when patient 2 had multiple skin lesions but before a diagnosis of monkeypox had been considered. The use of standard PPE may not have afforded sufficient protection against monkeypox, particularly if skin lesion debris containing virus had been disturbed and inhaled when bedsheets were changed.

Although patient 3 received postexposure vaccination before symptom onset, vaccination was >4 days after the most recent exposure to patient 2. The optimal timing for postexposure vaccination with Imvanex remains unknown, and the postexposure window period chosen for this incident was informed, in part, by that used during the US outbreak in 2003 (*6*). Patient 3 may have been vaccinated too late to prevent monkeypox.

During this incident, the risk to the public was determined to be very low because effective human-to-human transmission requires close contact with an infected person or virus-contaminated materials. Regardless, monkeypox is considered an HCID in England because it meets the UK criteria (*13*).

Monkeypox cases associated with travel to Nigeria have subsequently been detected in Israel (*14*) and Singapore (*15*). Although monkeypox is rare outside disease-endemic countries in Africa, this incident illustrates the need to be aware of monkeypox as a reemerging and travel-associated infection. Clinicians should consider a potential diagnosis of monkeypox early for patients with compatible symptoms and potential exposure risks, including recent travel to a disease-endemic country. In healthcare settings, implementation of appropriate infection prevention and control precautions as soon as monkeypox is suspected will help prevent secondary transmission.
